# Distinct and dementia‐related synaptopathy in the hippocampus after military blast exposures

**DOI:** 10.1111/bpa.12936

**Published:** 2021-02-24

**Authors:** Michael F. Almeida, Thuvan Piehler, Kelly E. Carstens, Meilan Zhao, Mahsa Samadi, Serena M. Dudek, Christopher J. Norton, Catherine M. Parisian, Karen L.G. Farizatto, Ben A. Bahr

**Affiliations:** ^1^ Biotechnology Research and Training Center University of North Carolina—Pembroke Pembroke NC USA; ^2^ U.S. Army Research Laboratory Aberdeen Proving Ground MD USA; ^3^ Neurobiology Laboratory National Institute of Environmental Health Sciences Research Triangle Park NC USA; ^4^ Present address: Center for Computational Toxicology and Exposure U.S. Environmental Protection Agency Research Triangle Park NC USA; ^5^ Present address: Faculty of Medicine Centre Imperial College London London UK

**Keywords:** Alzheimer‐type synaptopathogenesis, mild traumatic brain injury, NBDP, NCAM breakdown products, neurotrauma, synaptic decline

## Abstract

Explosive shockwaves, and other types of blast exposures, are linked to injuries commonly associated with military service and to an increased risk for the onset of dementia. Neurological complications following a blast injury, including depression, anxiety, and memory problems, often persist even when brain damage is undetectable. Here, hippocampal explants were exposed to the explosive 1,3,5‐trinitro‐1,3,5‐triazinane (RDX) to identify indicators of blast‐induced changes within important neuronal circuitries. Highly controlled detonations of small, 1.7‐gram RDX spherical charges reduced synaptic markers known to be downregulated in cognitive disorders, but without causing overt neuronal loss or astroglial responses. In the absence of neuromorphological alterations, levels of synaptophysin, GluA1, and synapsin IIb were significantly diminished within 24 hr, and these synaptic components exhibited progressive reductions following blast exposure as compared to their stable maintenance in control explants. In contrast, labeling of the synapsin IIa isoform remained unaltered, while neuropilar staining of other markers decreased, including synapsin IIb and neural cell adhesion molecule (NCAM) isoforms, along with evidence of NCAM proteolytic breakdown. NCAM_180_ displayed a distinct decline after the RDX blasts, whereas NCAM_140_ and NCAM_120_ exhibited smaller or no deterioration, respectively. Interestingly, the extent of synaptic marker reduction correlated with AT8‐positive tau levels, with tau pathology stochastically found in CA1 neurons and their dendrites. The decline in synaptic components was also reflected in the size of evoked postsynaptic currents recorded from CA1 pyramidals, which exhibited a severe and selective reduction. The identified indicators of blast‐mediated synaptopathy point to the need for early biomarkers of explosives altering synaptic integrity with links to dementia risk, to advance strategies for both cognitive health and therapeutic monitoring.

## Graphical abstract

Shockwaves produced by military explosives, which have been linked to enhanced dementia risk, cause selective reductions among synaptic markers in circuitries of the hippocampus, and a sharp decline in synaptic functionality corresponded with the reduced immunolabeling of synapses.The blast‐induced effects are evident in seemingly healthy neuronal fields, with the distinct synaptic compromise and early tau accumulation occurring without any indications of neuromorphological alterations or astroglial activation.The synaptopathy initiated by blast exposures may tip the balance towards the synaptic compromise associated with mild cognitive impairment and dementia, thus understanding blast‐induced changes in central synapses is vital for managing dementia risks later in life.

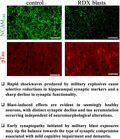

## 1 INTRODUCTION

Explosive blasts account for a majority of the injuries among wounded service members, as hundreds of thousands of veterans from wars of the 21st century are estimated to have experienced brain injuries caused by military or improvised explosives (1, 2, 3), and signs of neuropathology were found in postmortem brains from veterans that died years after blast exposures (4). Further alarming are the numbers of blast‐exposed individuals returning from war zones with no detectable physical injury or neuropathology, but who still suffer from persistent neurological symptoms including depression, headaches, anxiety, irritability, sleep disturbances, blurred vision, and memory problems. Delayed cellular responses occur due to blast exposure (5), suggesting that a subset of exposed individuals can have no apparent injury or symptoms, only to experience delayed effects to brain mechanisms and behavioral functions. Timely identification and proper managing of blast‐induced brain trauma are critical since traumatic brain injuries (TBIs) often worsen in clinical outcome within 48 hr if not treated appropriately (6). Those with blast‐induced neurotrauma and associated neurological intrusion, but without the typical neuropathology of a TBI, face a major challenge for diagnosis. Neuroimaging techniques are improving but have yet to achieve the level of sensitivity needed to detect subtle blast‐induced alterations that underlie lasting neurological impediments.

Previously, we exposed organotypic brain slice cultures to blast waves mediated by reproducible, spherical assemblies of the military explosive RDX (7). The blast intensity generated by the detonations was at a level that did not cause cell death, while causing synaptic vulnerability evident before overt neuropathology. As suggested, the mystery behind blast‐induced neurological complications when traumatic damage is undetected may be rooted in distinct alterations to synaptic integrity. Synaptic compromise may also underlie the cognitive deficits and other symptoms found among individuals with prolonged exposures to low‐level blasts from obstacle breaching, shoulder‐fired weapons, and related heavy weapons training (8, 9). In addition to war‐zone experiences and terrorist events, blast exposures that endanger the brain likely include those connected with construction sites and film productions that utilize explosives.

We hypothesize that, when exposed to rapid shockwaves created by military‐grade explosives including Composition C‐4 (91% RDX), brain areas involved in emotional‐laden memories and social behavior experience the types of neuronal changes found in cognitive disorders (10, 11, 12, 13). As noted by the previous studies, the hippocampus is a particularly vulnerable brain region at early stages of the disorders that include mild cognitive impairment (MCI) and dementia. Accordingly, we subjected rat hippocampal explants to detonations of highly controlled RDX spherical charges and assessed synaptic components and functional measures. Surprisingly, although military blast‐induced synaptopathy shared several features with MCI and Alzheimer‐type pathogenesis, one marker––synapsin IIa––was distinctly unaffected by explosive shockwaves as compared to the consequences of the neurodegenerative disorders. This study stresses the need to develop specific and sensitive imaging techniques to evaluate blast‐induced alterations in important neuronal networks.

## 2 RESULTS

**FIGURE 1:**
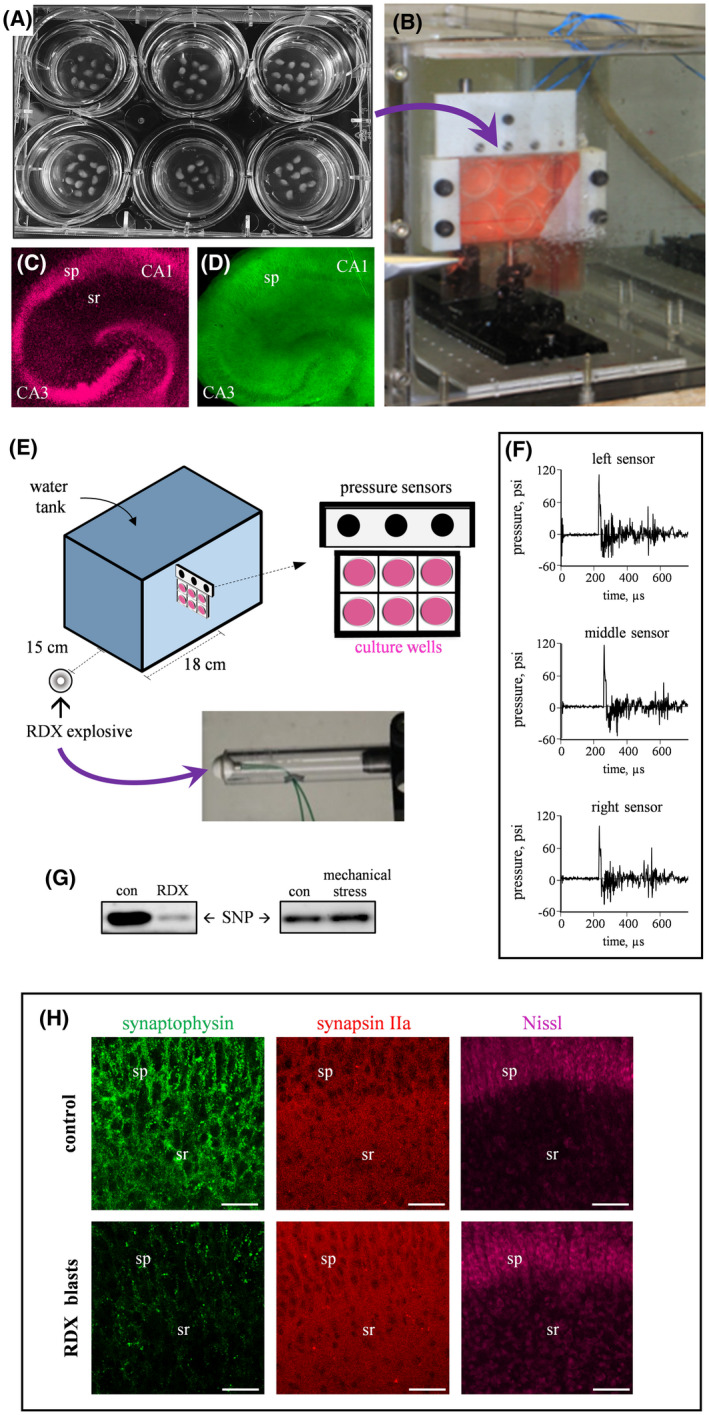
Military blast‐generated shockwaves lead to synaptic alteration in hippocampal explants. (A) Transverse slices of rat hippocampi were maintained on 3‐cm inserts for 18‐22 days in a six‐well culture plate. (B) The culture plate was sealed in serum‐free medium and clamped in a water‐filled chamber for military blast exposures. Control slice cultures exposed to the serum‐free conditions of the apparatus were assessed by Nissl (C) and synaptophysin staining (D), showing the stable maintenance of hippocampal subfields and associated dense neuropil (view‐field width: 1.5 mm). (E) Small spherical assemblies of RDX explosive were detonated outside the tank. (F) The shockwave propagated into the tank and was recorded as a time‐pressure profile by three sensors located above the culture plate. (G) Explants subjected to RDX detonations were assessed by immunoblotting for synaptophysin levels (SNP) and compared to control explants treated with identical conditions without blasts (con) or with mechanical stress produced by consecutive flexing protocols applied to the Biopore insert membrane. (H) Control and blast‐exposed explant cultures were fixed and subjected to synaptophysin, synapsin IIa, and Nissl staining (scale bars: 50 µm). sp, stratum pyramidale; sr; stratum radiatum

To test for neuronal changes resulting from military blast exposure, brain tissue in a six‐well culture plate (Figure 1A) was placed into a specialized chamber designed to withstand explosive shockwaves (Figure 1B). Transverse slices of hippocampus were used for the study, rapidly prepared under ice‐cold conditions and the explants positioned onto Biopore insert membranes for an extended culture period. Note that the hippocampal explants maintained native neuronal organization and synaptic density after three weeks in culture, even after being subjected to control conditions in the experimental setup (Figure 1C,D). The established blast paradigm consisted of detonating a spherical assembly of the military explosive RDX positioned at a precise distance from the wall of the blast chamber (Figure 1E). The detonation created a shockwave that propagated into the water‐filled chamber and registered similar pressure profiles at three sensors located just above the culture plate (Figure 1F), producing a peak pressure of 109.4 ± 3.05 psi (mean ± SEM) followed by a delayed reflected pressure wave of approximately 50 psi.

Across the treated explant samples, detonations of RDX spherical charges produced a 60‐80% decline in the synaptic protein synaptophysin as compared to control hippocampal slices treated with identical conditions in the constructed chamber but without blast exposures (Figure 1G, left; see expanded blot images and load control in Figure S1 of Supporting Information). An additional control was done since some levels of traumatic injuries have been suggested to involve neurite deformation, including axonal stretching, as part of a pathogenic mechanism toward neuronal deterioration (14, 15). Thus, to test whether the synaptic decline reported in the present study is due to neuronal deformation from shockwave‐induced distortion of the culture insert, we applied repeated flexing stress to the culture insert membrane. Such mechanical stress and flexing of the explant support membrane did not alter the level of synaptophysin (Figure 1G, right; also see Figure S1 of Supporting Information). The synaptic marker immunoreactivity measures after mechanical stress application were 98.4 ± 11.8% of control explants. Interestingly, the synaptopathy produced by the RDX blasts included significant reduction of synaptophysin staining in the CA1 dendritic field, whereas synapsin IIa immunolabeling was unaffected by the explosive blasts (Figure 1H). As shown, the shockwave intensities produced by the 1.7‐gram RDX charges sharply decreased punctate labeling of synaptophysin in both the pyramidal field and associated dendritic zone, and this evident synaptopathy occurred in the absence of neuronal loss or morphological alterations assessed with Nissl staining.

**FIGURE 2:**
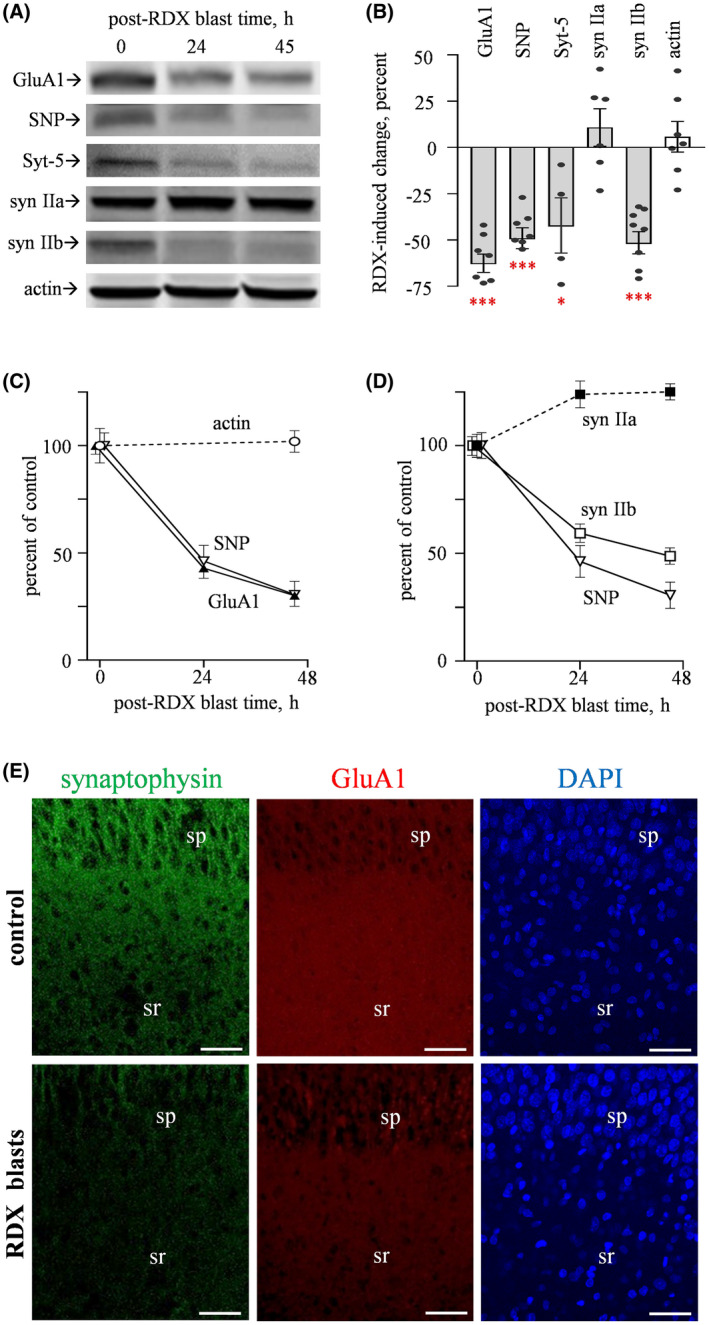
Blast waves from RDX spherical charges induce distinct synaptic vulnerability. (A) Hippocampal explants were subjected to three consecutive RDX detonations, then maintained in culture for 24‐45 hr, and subsequently assessed for GluA1 (n = 8), synaptophysin (SNP; n = 8), synaptotagmin‐5 (Syt‐5; n = 4), synapsin IIa (syn IIa; n = 6) and IIb (syn IIb; n = 9), and actin (n = 7) as a load control, with comparisons to explants treated with identical conditions without blasts (0 hr). (B) For the immunostained proteins, integrated optical densities were measured to determine blast‐induced changes as compared to respective measures from control explants (means ± SEM). *p < 0.05; ***p < 0.001. (C and D) Across post‐blast times, mean immunoreactivities were normalized to respective data from three control sets of pooled explants that received identical conditions without blasts and harvested 45 hr later. The following results were from one‐way ANOVA tests: SNP, *p* = 0.0001 (n = 11); GluA1, *p* < 0.0001 (n = 11); syn IIb, *p* < 0.0001 (n = 7). (E) Slice cultures treated with or without RDX blasts were fixed and subjected to synaptophysin and GluA1 immunolabeling, followed by DAPI counterstaining (scale bars: 50 µm). sp, stratum pyramidale; sr; stratum radiatum

To further address distinctive vulnerabilities among synaptic markers, their measures were determined over a period of two days after the RDX detonations (Figure 2A; see expanded blot images in Figure S2 of Supporting Information). Compared to control explants with equal post‐treatment time after incubation in the experimental chamber, the immunoblot data found significant blast‐induced reductions in synaptophysin (*p* < 0.001), synapsin isoform IIb (*p* < 0.001), synaptotagmin‐5 (*p* < 0.05), and in the AMPA‐type glutamate receptor subunit GluA1 (*p* < 0.0001), whereas no changes were found for synapsin IIa or the housekeeping protein actin (see Figure 2A,B). As shown in Figure 2C, synaptophysin (*p* = 0.001, Tukey's post hoc analysis) and GluA1 levels (*p* < 0.0001) were reduced by > 50% 24 hr subsequent to consecutive blasts, and by nearly 75% at the 45‐h post‐blast time (p ≤ 0.0001). In contrast to the unaltered synapsin IIa, the IIb isoform exhibited a time‐dependent reduction after the explosive shockwaves (Figure 2D; *p* < 0.0001 at 24 and 45 hr), having a slightly slower decline as compared to those displayed by synaptophysin and GluA1. Regarding the latter synaptic markers, note that the RDX blasts caused a 60‐80% reduction in punctate neuropilar staining of the presynaptic protein, and the postsynaptic GluA1 labeling exhibited a marked decrease in the same CA1 dendritic zone (Figure 2E), with no obvious change in DAPI‐labeled nuclei after the detonations.

**FIGURE 3:**
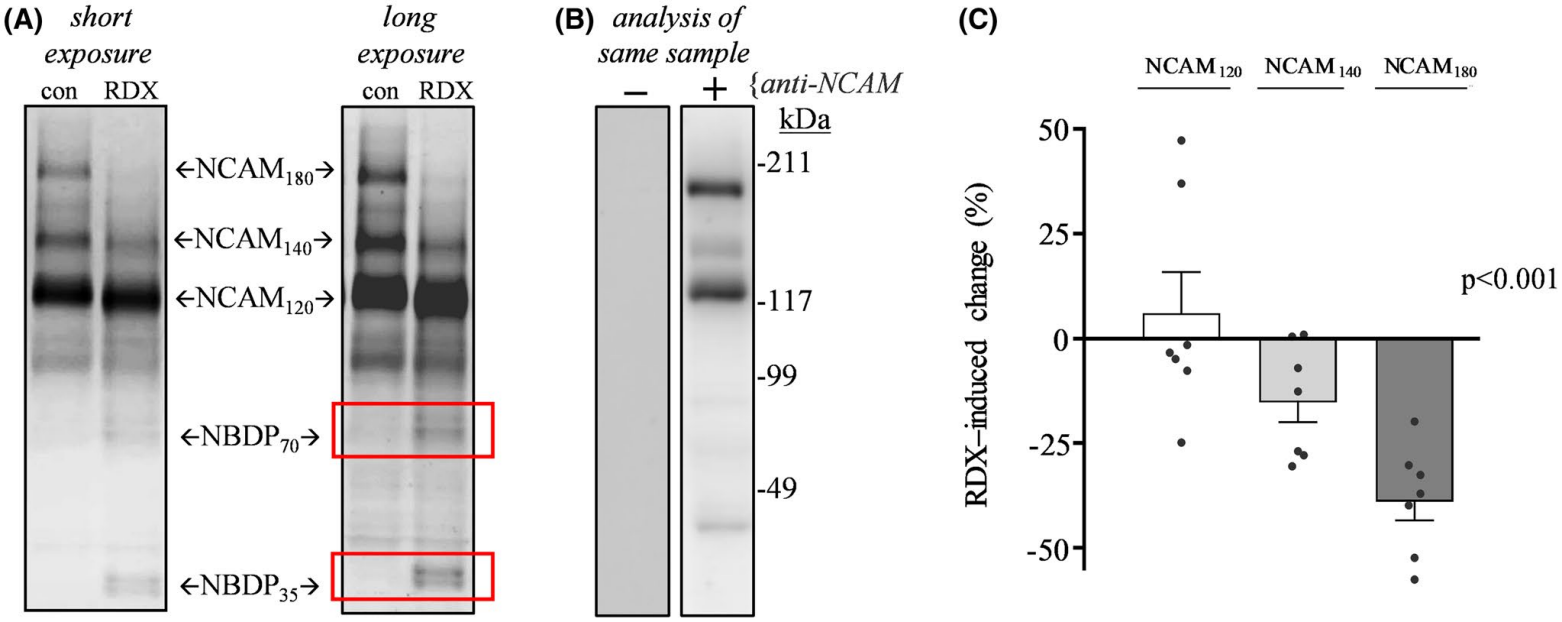
NCAM isoforms exhibit distinct blast‐induced effects. (A) Three NCAM isoforms were assessed in cultured hippocampal slices that were exposed to consecutive RDX detonations and compared to control explants treated identically without blasts (con). The immunoblots were also analyzed with longer chemiluminescent exposure times, revealing NCAM breakdown products (NBDPs, red boxes). (B) The NCAM isoforms were selectively labeled by anti‐NCAM antibody as there was no detectable labeling of 120‐, 140‐, or 180‐kDa proteins in the blot strip incubated with non‐selective IgG during the immunoblotting protocol. (C) NCAM immunoreactivity measures (n = 7) were compared to their respective levels in control explants (mean percent change ± SEM), and the blast‐induce effects were determined to be significantly different among the isoforms (Kruskal‐Wallis test: *p* < 0.001)

Those synaptic markers decreased by RDX blasts have also been found reduced in Alzheimer's disease (AD) brains (16), thus we further addressed the hypothesis that blast shockwaves cause AD‐type synaptic alterations by evaluating synaptic adhesion molecules with links to age‐related synaptic decline and deficits in brains of those with AD (17, 18, 19, 20). We assessed neural cell adhesion molecule (NCAM) species, and the blast‐exposed hippocampal explants exhibited selective vulnerability among them (Figure 3A). The NCAM isoforms of 120, 140, and 180 kDa were specifically labeled by the Millipore anti‐NCAM antibody since no immunolabeling was evident when the same blot strip was first incubated with non‐selective IgG (Figure 3B). Immunoreactivity levels of the three isoforms were measured and compared to their respective levels in control explants, indicating a substantial reduction in NCAM_180_ (*p* < 0.001), while NCAM_140_ (*p* = 0.0283) and NCAM_120_ (*p* = 0.565) exhibited a much smaller decline or no reduction, respectively (Figure 3C). Figure S1 of the Supporting Information shows that immunoblot levels of the NCAM isoforms were not affected by repeated mechanical stress applied to the culture inserts. Also, it is of interest that with longer exposure time to visualize low‐staining bands, the same samples displaying blast‐induced NCAM reduction had breakdown products revealed that are generated by the shockwaves: a 65/70‐kDa doublet species (NBDP_70_) and a 30/35‐kDa doublet (NBDP_35_; see red boxes in Figure 3A).

**FIGURE 4:**
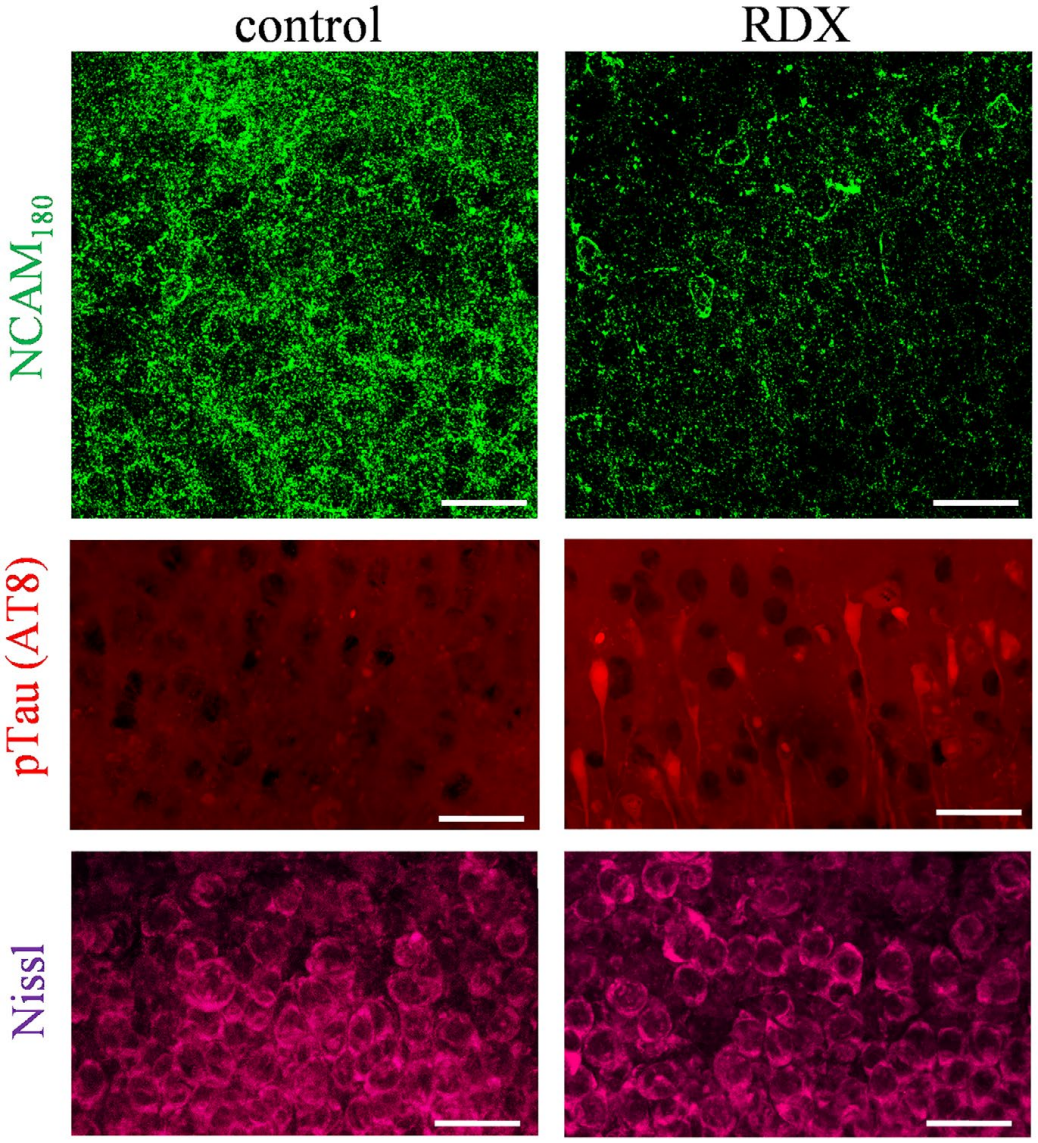
Blast‐induced reduction of neuropilar NCAM_180_ labeling was associated with pathological tau staining. Blast‐exposed and control explants were fixed and immunolabeled for the vulnerable NCAM_180_ isoform (top panels; scale bar: 25 µm) and for phosphorylated tau (pTau) using the AT8 monoclonal antibody (middle panels; scale bar: 50 µm). Counterstaining by Nissl protocol found no obvious indications of neuronal alterations (bottom panels; scale bar: 25 µm). sp, stratum pyramidale; sr; stratum radiatum

Further substantiation of the NCAM immunoblot data was found with immunocytochemical analysis using NCAM_180_‐specific antibodies. The anti‐NCAM_180_ staining determined dramatic reduction of punctate immunolabeling in the hippocampal neuropil of blast‐exposed explants (Figure 4, top panels). Also, as indicated in previous figures, the Nissl counterstaining confirmed the absence of any obvious neuronal alterations (Figure 4, bottom). The middle panels of Figure 4 show that blast‐induced synaptic decline is associated with pre‐tangle tau staining in hippocampal zones, thus supporting our hypothesis that early dementia‐related pathogenesis is initiated by blast exposures. Note that NCAM alterations were previously reported to correlate with pathogenic tau load in the AD human brain (20). The RDX‐exposed and control explants were immunostained with the AT8 monoclonal antibody against phosphorylated tau at Ser202 and Thr205. As shown in the micrographs, the detonations caused stochastic labeling of both CA1 pyramidal neurons and their processes, similar to the diffuse tau pathology identified in hippocampal CA1 of military veterans that died 1‐2 years after exposure to improvised explosive blasts (4). It is also noteworthy that across the hippocampal explant treatment groups, the extent of synaptic marker decline correlated with measures of AT8‐positive tau levels (*R* = –0.667; *p* < 0.01).

**FIGURE 5:**
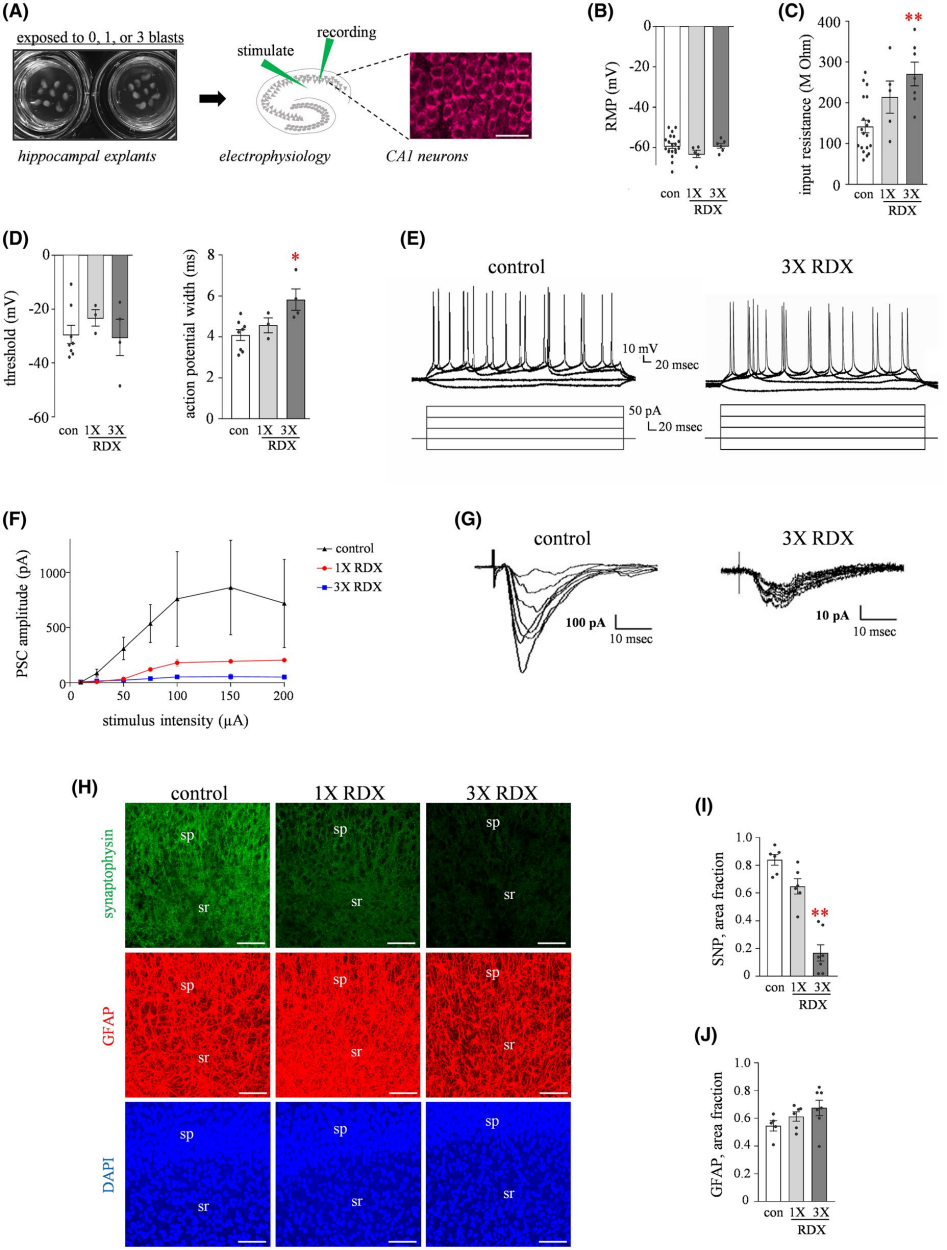
Blast‐induced functional changes in CA1 neurons. (A) Hippocampal slice cultures were subjected to detonations of 1‐3 RDX spherical charges then assessed 24‐48 hr later using whole‐cell patch clamp to record intrinsic properties. (B) Control explants that received identical conditions without blasts (con; n = 20) and the RDX‐treated explants (n = 11) were assessed for resting membrane potential in pyramidal neurons (RMP; means ± SEM). (C) In control (n = 19) and blast‐exposed explants (n = 12), the input resistance was also measured in the neurons (one‐way ANOVA: *p* = 0.0013; Bonferroni test: **p < 0.001). (D) Additional measures found that action potential firing threshold was unchanged across the explant treatment groups (left graph), while action potential half‐width exhibited a 45% increase after multiple blasts compared to controls (right graph; ANOVA *p* = 0.0157; Bonferroni test: *p = 0.014). (E) Representative traces are shown for control explants and those exposed to three consecutive blasts. (F) The blast exposures reduced the amplitude of evoked postsynaptic currents (PSC) as well in response to varied stimulation intensities to the Schaffer collateral inputs. (G) Note the 10‐fold difference in amplitude scales between the two sets of representative traces. (H) A subset of the control explants (n = 6) and the RDX‐exposed explants (n = 12) were subsequently stained for synaptophysin, GFAP, and DAPI (scale bars: 50 µm). (I) Resulting from image analysis of confocal micrographs, synaptophysin immunostaining in CA1 revealed blast‐induced progressive decline in the neuropil (ANOVA *p* < 0.01; Tukey test: **p = 0.0057). (J) In the same assessed area of the stratum radiatum, GFAP immunostaining showed no indication of early astroglial activation by area fraction analysis. sp, stratum pyramidale; sr, stratum radiatum

Finally, we tested whether the effects of RDX blasts influence functional integrity related to synaptic transmission (Figure 5A). Using whole‐cell patch clamp, the assessed CA1 neurons were seemingly healthy after the detonation of spherical charges since resting membrane potentials (Figure 5B) and action potential firing thresholds were unaffected (Figure 5D, left graph). However, other distinct measures were altered, including a dose‐dependent rise in input resistance (Figure 5C; one‐way ANOVA: *p* = 0.0013) as compared to control slices treated identically but without blast exposures. The consecutive RDX blasts also significantly increased the action potential half‐width (Figure 5D, right graph; representative traces in 5E). More indicative of synaptic compromise was the reduction in evoked postsynaptic current size assessed by whole‐cell voltage clamp in response to increasing stimulation intensity applied to Schaffer collateral inputs (Figure 5F,G; Dunnet tests: *p* < 0.05 for control explants compared to both single and triple‐blast groups). After the electrophysiological recordings the hippocampal explants were fixed and immunostained for synaptophysin, revealing the blast‐induced progressive decline in the synaptic labeling in the neuropil (Figure 5H—top, and Figure 5I; *p* < 0.01). DAPI‐labeled nuclei in the analyzed dendritic field showed no blast‐induced changes (Figure 5H—bottom). The DAPI images assessed as area fraction resulted in 35.6 ± 4.6% in control explants and 35.0 ± 2.4% in explants exposed to three consecutive blasts. Thus, the decline in synaptic functionality corresponded with reduced labeling of synapses, but without any obvious cellular loss. In addition, double‐staining for glial fibrillary acidic protein (GFAP) alongside synaptophysin found that during the induced synaptopathy the astrocytic network remaining unchanged (Figure 5H—middle, and Figure 5J), with no appreciable change in the GFAP area fraction assessment in the stratum radiatum.

## 3 DISCUSSION

The effects of explosive blasts on the central nervous system are an increasingly common cause of persistent and often debilitating neurological problems among both military service members and civilians. Our findings reveal that detonations of military explosives produce distinct alterations in neurocircuitries of the hippocampus, a higher brain center involved in episodic memory encoding and social behavior (21, 22). The early indications of neuronal compromise include the downregulation of synaptic components as well as reduced synaptic transmission measured in the CA1 dendritic zone. The electrophysiological alterations have the potential to disrupt the encoding or interpretation of complex information relayed to hippocampal connections, involving networks affected by a variety of neurological and psychiatric disorders. Just as neurological complications from blast exposures often occur in the absence of detectable brain damage, a significant finding in this study is that the synaptopathy determined after explosive blasts arises ominously in the dense hippocampal neuropil, even though neuronal loss, morphological alterations, or changes in resting membrane potential were not evident.

We could perform this study because the precise assembly of 1.7‐gram RDX spherical charges allowed reproducible explosive shockwaves to be directed toward brain explants. The latter were prepared with an organotypic culture technique that stably maintains the organization and synaptic complexity of the hippocampal region that is involved in high ‐order network processing. Early and distinct synaptic disruption was indicated by a decrease in the evoked postsynaptic current amplitude after a single RDX blast, whereas the threshold for action potential induction was unchanged. Severe reduction in the evoked responses occurred after multiple blasts, and similarly progressive changes in action potential width and input resistance were found. Corresponding with the functional compromise, synaptopathy was also indicated by the blast number‐dependent and time‐dependent loss of synaptophysin, synapsin IIb, and a synaptotagmin, presynaptic proteins known to be downregulated as part of the early synaptic deterioration of AD‐type proteinopathy (13, 16, 23). In a related study, mice exposed to detonations of trinitrotoluene (TNT; shockwave peak overpressure force of 2.5 psi) exhibited significant losses of synaptophysin in the hippocampus and temporal cortex along with corresponding impairment in spatial working memory (24). The study also found that a neuroprotective drug approved for type II diabetes (exendin‐4 polypeptide) prevented the induction of both synaptophysin reduction and cognitive impairment. Across the present and previous studies, the decline in synaptic vesicular components may be related to findings that TBI disrupts synaptic vesicle distribution in the hippocampus (25), thereby promoting the weakening of neurotransmission and potential cognitive impairment.

While the presynaptic proteins listed above were significantly attenuated in the hippocampal explants, the military blast exposures had little effect on the presynaptic isoform of NCAM (NCAM_140_) and no measurable detriment targeting synapsin IIa. Thus, in particular, the synapsin IIa and IIb isoforms clearly exhibit dissimilar responses with regards to blast‐induced synaptopathy, perhaps related to the former isoform being linked to vesicular reserve pool regulation (26) versus the latter involved in the formation of presynaptic terminals (27). It is also not unexpected that some synaptic markers would exhibit little or no deterioration in response to small RDX explosives, especially given the subtle nature of the early synaptopathy in the absence of overt neurodegeneration. Note that the difference in responsiveness is not due to synapsin IIa being less vulnerable to pathogenic events than synapsin IIb, since the IIa isoform also exhibits severe reductions in models of neurotoxin exposure (28), age‐related lysosomal perturbation (23), and Alzheimer's and Parkinson's diseases (29). In fact, reduced levels of synapsin IIa strongly correlated with the extent of memory impairment across AD transgenic mice with varying stages of protein accumulation pathology (29). In light of the distinction between the two synapsins, as well as the growing evidence that mild brain injuries are linked to dementia emerging later in life, it is important to point out that selective positron emission tomography (PET) ligands targeting the two synapsin isoforms may be able to discern between early effects and subsequent levels of pathology as part of identifying blast‐related risk factors. The localization of military blast‐induced synaptopathy in the brain may lead to the furthering of MRI and PET techniques being developed (14, 30, 31), to facilitate sensitive detection of those compromised neurocircuitries responsible for behavioral deficits.

Regarding postsynaptic markers, GluA1 was previously found to decline in a blast dose‐dependent manner (7). In the present study, levels of the glutamate receptor subunit were sharply lowered by blast exposures, following a similar decay profile as expressed by synaptophysin. Additional synaptic alterations involve NCAM_180_ and NCAM_140_ whose levels declined due to the RDX detonations. Interestingly, just as the postsynaptic NCAM_180_ exhibits the most blast‐mediated vulnerability, such isoform‐selective reduction has been reported in aged mice (17, 18), in the hippocampus of chronically stressed mice in which the reduction in NCAM expression was linked to cognitive impairment (18), and also appears to occur in the distal hippocampus after severe controlled cortical impact injury (32). Further related to the neurological complications stemming from blast injuries, NCAM deletion was found to increase aggression and alter emotionality in a stress‐treated animal model (33). In addition, a previous report using a distinct disease model (diabetic rat) found corresponding reductions in hippocampal GluA1 and NCAM expression levels that were accompanied by impaired synaptic currents mediated by the glutamatergic channels (34). Thus, beyond the alterations in adhesion molecules with links to AD and age‐related cognitive decline (17, 18, 19, 20), the military blast‐induced changes in synaptic adhesion components may also be involved in the resulting disturbances in cognition and emotional behaviors.

NCAM species add to the list of synaptic proteins that are reduced by RDX detonations and are also known to display substantial declines in AD patients and animal models (11, 16, 35). While moderate and severe TBI have been previously associated with increased dementia risk, it is of particular interest that mild TBI without loss of consciousness was recently linked to dementia as well, with more than a two‐fold increase in the risk of dementia diagnosis (36). Thus, the fact that RDX‐exposed explants exhibit similar tau pathology as found in veterans with mild TBI drew our attention to the human results related to blasts from improvised explosive devices. As described, RDX blasts led to tau pathology in stochastic patterns of CA1 neurons and processes extending into the neuropil, and the increase in pathogenic tau measures correlated with synaptic marker decline. Blast exposures in a rat shock tube model also resulted in neuronal buildup of phosphorylated tau and its somatodendritic redistribution (37). While phosphorylated tau accumulation is considered the primary proteinopathy in certain brain injuries including chronic traumatic encephalopathy, the current study did not test for changes in the Aβ peptide. However, both intra‐axonal Aβ accumulation and increased phosphorylated tau levels were previously found in a controlled cortical impact model using AD transgenic mice (38). As NCAM is an element of the distinct synaptopathy induced by military blasts, a study of AD cases is of interest since they found reduced measures of an NCAM marker that correlated with pathogenic, hyperphosphorylated tau levels (20). The correlative result was identified in the entorhinal cortex of the human brains, and this region is a vital component of the hippocampal memory system by providing the first projection of the trisynaptic circuit and distributing hippocampal signals across cortical areas.

The need for early biomarkers of blast injuries cannot be stressed enough in order to identify those initial neuronal alterations that may tip the scales toward dementia. The distinct synaptopathy in blast‐exposed hippocampal tissue has resemblances with AD‐type neuronal compromise. Models of the more severe designation of TBI have been found to produce potential biomarkers including noncoding microRNAs, with the inflammation‐associated microRNA miR155 increased after lateral fluid‐percussion injury in rats and in the post‐TBI human cortex with evident association with activated astrocytes (39). In contrast, the subtle, early effects following small RDX detonations were not associated with astroglial activation, nor was NCAM_120_ affected which is expressed mainly by glia in the CNS. Axonal swelling from cytoskeletal disruption and transport interruption is also an indicator of TBI, and the controlled cortical impact model resulted in a prolonged and multiphasic period of axonal swelling (40). Such axonopathy and related transport deficits are characteristic of a non‐impact model of mild TBI (41) as well as early AD pathogenesis (12). Note that the cytoskeletal disruption indicated by a 145‐kDa αII‐spectrin proteolytic product (SPP), a putative biomarker of diffuse axonal injury, was found across moderate and severe TBI in humans and mice (41, 42, 43), as well as in hippocampal neuropilar patterns after brief excitotoxicity (44, 45) and of aging rodents (46, 47). Again, contrary to these indicators, the RDX blast model did not exhibit SPP in association with early synaptic alterations (7), and repetitive shock tube blasts did not cause SMI‐32–positive axonal damage in hippocampal cultures (48). Thus, as previously suggested, different pathological phenotypes that influence axonal and synaptic integrity occur across the varying levels of brain injury and even due to events of brain aging, the latter being related to the major risk factor of AD.

Military blast shockwaves lead to a level of synaptic pathology that is more subtle than mild TBI, perhaps explaining the enhanced dementia risk by initiating Alzheimer‐type neuronal compromise. Thus, a key biomarker is one that can identify early synaptic alterations specifically mediated by blast exposure, especially those related to dementia‐type synaptopathology. The ability to detect NCAM breakdown products would indeed enhance imaging sensitivity for the important long‐term monitoring of synaptic pathology. The generation of proteolytic fragments by blast exposure is especially relevant due to NCAM alterations having been implicated in AD as well as psychiatric issues including bipolar disorders, depression, and anxiety disorder (49). Note that pathogenic proteolysis of NCAM has been previously reported (50, 51), and the NCAM proteolysis was linked to behavioral pathology as well (51). Early detection of measurable synaptopathy may lead to vital improvements in diagnoses, the treatment of recurring neuropsychiatric impediments, and reducing the risk of developing dementia later in life, as the increased risk is likely rooted in the disruptions of synaptic communication instigated by blast exposures.

## 4 MATERIALS AND METHODS

### 4.1 Organotypic hippocampal slice cultures

Sprague‐Dawley rat litters (Charles River Laboratories; Wilmington, Massachusetts) were housed and treated in accordance with the recommendations from the Guide for the Care and Use of Laboratory Animals from the National Institutes of Health, and following an approved protocol from the Institutional Animal Care and Use Committee of the University of North Carolina–Pembroke. Hippocampal slice cultures were prepared from 12‐day‐old rats as previously described (13, 23). The transverse slices were cut from pre‐cooled hippocampi and briefly placed in ice‐cold buffer containing 124 mM NaCl, 3 mM KCl, 2 mM CaCl_2_, 4 mM MgSO_4_, 1.25 mM KH_2_PO_4_, 26 mM NaHCO_3_, 10 mM D‐glucose, and 2 mM ascorbic acid. Groups of eight to nine slices were then quickly distributed on the Biopore PTFE membrane of each Millicell‐CM culture insert (Fisher Scientific; Pittsburgh, Pennsylvania) which was in contact with culture medium containing 50% BME, 25% Earl's balanced salt solution, 25% regular horse serum, and the following concentrations of supplements: 136 mM glutamine, 40 mM glucose, 0.5 mM ascorbic acid, 20 mM HEPES buffer (pH 7.3) 1 mg/L insulin, 5 units/ml of penicillin, and 5 mg/L streptomycin. Medium was changed every 2‐3 days, and the surfaces of the slices were exposed to humidified air plus 5% CO_2_ at 37 °C for an 18‐22‐day maturation period before experiments were conducted.

### 4.2 Primary blast

Hippocampal slices from a six‐well plate were sealed in an air‐tight containment filled with serum‐free media (SFM; horse serum was replaced with HEPES‐buffered saline). Next, the plate was clamped in a vertical position using a rig that was then secured within a 37°C water‐filled chamber, 18 cm from the front inner wall. The vertical position directs the blast to the surface of the tissue slices, thus minimizing lateral stress on adhesion between the brain tissue and culture insert membranes. Reproducible assemblies of RDX spherical charges (1.7 g) were used to produce an explosive blast 15 cm from the chamber's outer wall, creating a well‐defined air shockwave that propagates into and through the chamber's volume of water (7, 15). Control slices in a similar six‐well plate received mock treatment of equal submersion time in the SFM containment. All detonations were performed by Army Research Laboratory personnel in a blast facility at Aberdeen Proving Ground, Maryland. The slice cultures were exposed to 1‐3 detonations of the RDX spheres approximately 4 min apart, then quickly returned to normal culture conditions.

### 4.3 Immunoblot assessments

In order to obtain adequate protein amounts to assess immunoreactivity measures of multiple markers, and also to importantly reduce the variance that can occur across single explants extracted from tissue spanning the septal to temporal poles of hippocampi, each immunoblot sample consisted of seven to nine slice cultures that were gently removed from the insert membrane 1‐2 days after the blast events or comparable control conditions. Thus, each group of harvested explants represents an *n* value for an experimental condition. They were rinsed with ice‐cold isosmotic buffer containing protease inhibitors, homogenized in lysis buffer, and protein concentrations determined using a Pierce BCA protein assay (Thermo Fisher Scientific; Waltham, Massachusetts). Equal protein aliquots were denatured for 5 min at 100°C, separated by SDS‐PAGE, and transferred to nitrocellulose membranes (Bio‐Rad; Hercules, California). Next, blot membranes were blocked with 5% non‐fat dry milk for 1.5 hr at room temperature, followed by incubation with primary antibodies listed in Table S1 of the Supporting Information. They were against GluA1, synapsin II and NCAM (from Millipore), synaptophysin (Boehringer Mannheim), synaptotagmin V (BD Biosciences), and Actin (Sigma‐Aldrich). Anti‐IgG‐alkaline phosphatase conjugates and anti‐IgG‐horseradish peroxidase conjugates were used for the secondary antibody step, and antigen staining and image development involved chemiluminescence protocols using the GE Amersham AI600RGB imager. Immunostained bands were scanned at high resolution to determine integrated optical density (IOD) with BIOQUANT software (R & M Biometrics; Nashville, Tennessee). Within blots, each IOD value was normalized to the percent of mock treatment control samples, and the means ± SEM were compared using Prism software (GraphPad; San Diego, California).

### 4.4 Immunohistochemistry and confocal microscopy

A group of cultured hippocampal slices fixed in 4% paraformaldehyde were rinsed in PBS, blocked with 5% BSA in PBS containing 0.1% TX100 for 1.5 hr. Next, the explants were permeabilized with 0.3% triton‐x 100 in 0.1 M PBS for 30 min at room temperature, then blocked in 5% BSA in 0.1 M PBS containing 0.1% triton‐x 100 for 1.5 hr. The tissue was then incubated overnight with antibodies listed in Table S1 of the Supporting Information, against synaptophysin (Abcam), synapsin IIa (Santa Cruz Biotechnology, Dallas, Texas), NCAM_180_ (Abcam), GluA1 (Millipore), GFAP (Sigma‐Aldrich), and phosphorylated tau (AT8; Thermo Fisher Scientific). Immunolabeling was detected with Alexa Fluor‐488 or Alexa Fluor‐568 tagged secondary antibodies (Molecular Probe, Thermo Fisher Scientific). The specificity of fluorescent immunostaining for each antibody was confirmed by omission of the primary antibody, using consistent imaging parameters as per normal protocols. Routinely determined were negative images in the confocal channel for synaptic markers or for the glial marker GFAP in the experimental run with adjacent tissue treated with primary antibodies. At the end of the staining process, all slices were counter‐stained with DAPI (4′6‐diamidino‐2‐phenylindole; Vector Laboratories) or with a NeuroTrace fluorescent Nissl stain (Thermo Fisher Scientific). Stained hippocampal slice explant images were viewed with a Nikon C2 point‐scanning confocal microscope, captured and analyzed using NIS‐Elements Advanced Research software (Nikon Instruments Inc.).

### 4.5 Electrophysiology

Organotypic cultures were recorded while incubated in artificial cerebral spinal fluid (in mM): 124 NaCl, 2.5 KCl, 2 MgCl_2_, 2 CaCl_2_, 1.25 NaH_2_PO_4_, 26 NaHCO_3_, and 17 D‐glucose bubbled with 95% O_2_ with 5% CO_2_. Whole‐cell recordings were made from CA1 pyramidal neurons. Glass borosilicate pipettes were filled with a potassium gluconate internal solution (in mM) 120 K‐gluconate, 10 KCl, 3 MgCl_2_, 0.5 EGTA, 40 HEPES, 2 Na_2_‐ATP, 0.3 Na‐GTP, pH 7.2), with a tip resistance between 3 and 4.5 MOhms. Data were collected using Clampex 10.4 and analyzed using Clampfit software (Axon Instruments). Series and input resistances were monitored by measuring the response to a 10‐mV step at each sweep and cells were included for analysis if <20% change in series and input resistance. Cell health was assessed by resting membrane potential measures, and a measure higher than −45 mV was an established criteria to exclude a small number of cells in each treatment group. Cells with an input resistance >550 MΩ were excluded as this input resistance is typical of non‐pyramidal neurons in area CA1. Whole‐cell recordings were performed in voltage‐clamp mode, and postsynaptic currents (PSCs) were evoked with a bipolar cluster electrode (FHC Inc.; Bowdoin, Maine) that was placed in the stratum radiatum. To assess excitatory transmission, postsynaptic currents were measured at increasing stimulation intensities. To determine action potential threshold, whole‐cell recordings were performed in current‐clamp mode. Current pulses of 180 ms in 0.2‐nA steps were delivered and the membrane potential at which the cells first fired action potentials were measured. Action potential width was calculated by measuring the width at the base of the action potential initiation. Statistical analyses were performed using GraphPad Prism software, and significance was calculated using an alpha level of 0.05.

## CONFLICT OF INTEREST

The authors declare that they have no competing interests. The funding agencies had no role in study design, data collection and analysis, or decision to publish.

## AUTHOR CONTRIBUTIONS

M.F.A., T.P., S.M.D., and B.A.B. designed research; M.F.A., K.E.C., M.Z., M.S., C.J.N., K.L.G.F., and B.A.B. performed research; M.F.A., T.P., K.E.C., M.Z., M.S., S.M.D., C.J.N., K.L.G.F., and B.A.B. analyzed data; M.F.A., S.M.D., C.M.P., and B.A.B. wrote the paper; and B.A.B. supervised the entire project.

## Supporting information

Supplementary Material

## Data Availability

The data that support of this study are available from the corresponding author upon reasonable request.
