# Supplementary material for: Distinct and dementia‐related synaptopathy in the hippocampus after military blast exposures
Source: Brain Pathol. 2021 Feb 24;31(3):e12936. doi: 10.1111/bpa.12936 (PMC8412116; doi:10.1111/bpa.12936)

Almeida et al., Supplementary Table S1

Primary antibodies used to do immunoblot assessments

**Antibody against**	**host species**	**company (item)**
GluA1	rabbit	Millipore (ABN241)
synapsin II	rabbit	Millipore (574779)
NCAM	rabbit	Millipore (AB5032)
synaptophysin	rabbit	Boehringer Mannheim (SY38)
synaptotagmin V	mouse	BD Biosciences (612284)
actin	rabbit	Sigma-Aldrich (A5060)

Primary antibodies used to do immunohistochemistry and confocal microscopy

**antibody against**	**host species**	**company (item)**
GluA1	rabbit	Millipore (ABN241)
synapsin IIa	rabbit	Santa Cruz Biotechnology, (sc-36086)
$NCAM_{180}$	rabbit	Abcam, (ab75813)
synaptophysin	mouse	Abcam, (ab8049)
AT8	mouse	Thermo Fischer Scientific, (MN1020)
GFAP	rabbit	Sigma-Aldrich, (#G3893)

Almeida et al., Supplementary Figure S1

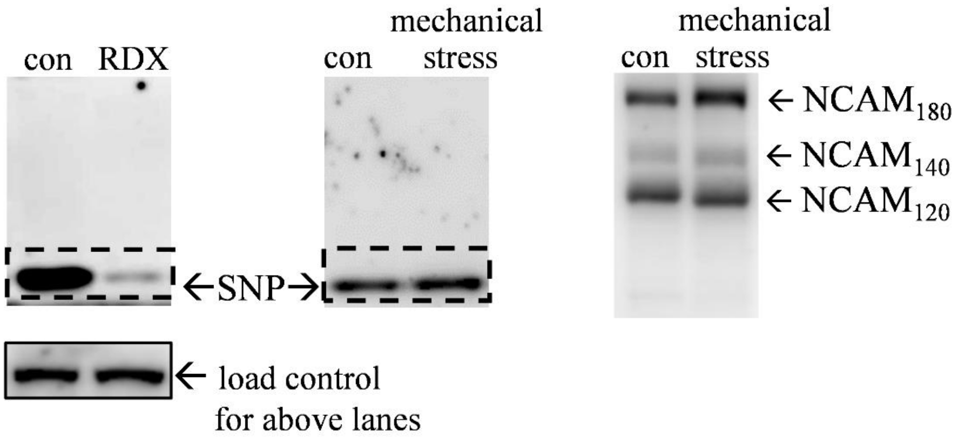

Almeida et al., Supplementary Figure S2

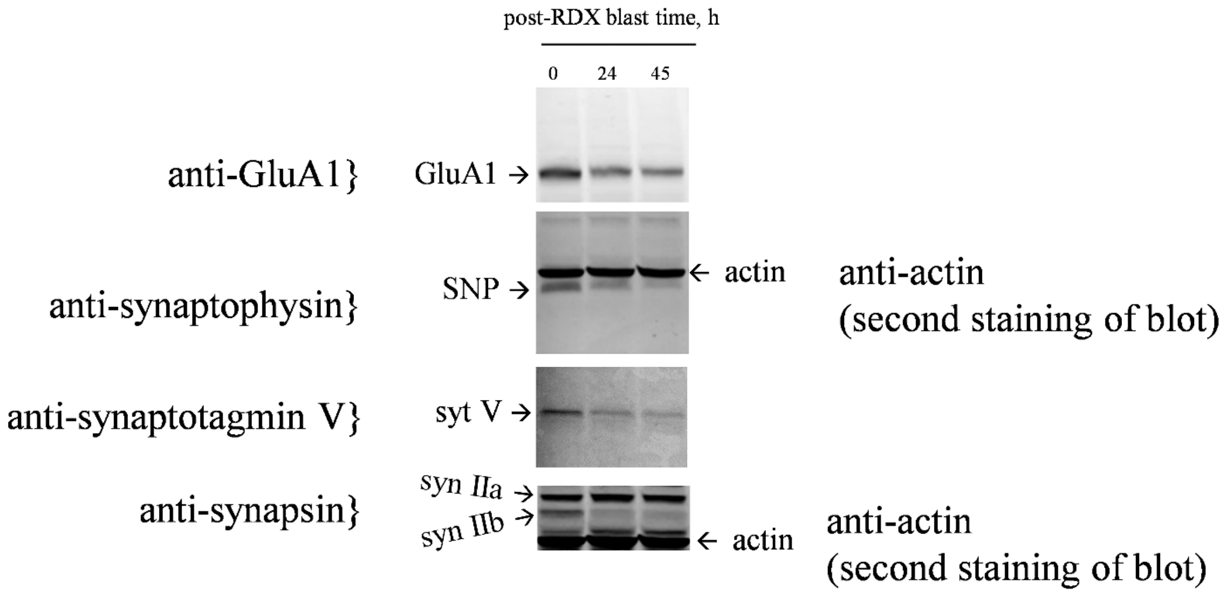

Supplement: Supplementary file 1 — Supplementary Material [file BPA-31-e12936-s001.pdf]
